# Somatic Comorbidities and Cardiovascular Safety in Ketamine Use for Treatment-Resistant Depression

**DOI:** 10.3390/medicina57030274

**Published:** 2021-03-16

**Authors:** Joanna Szarmach, Wiesław Jerzy Cubała, Adam Włodarczyk, Maria Gałuszko-Węgielnik

**Affiliations:** Department of Psychiatry, Faculty of Medicine, Medical University of Gdansk, 7 Dębinki St. Build. 25, 80-952 Gdansk, Poland; cubala@gumed.edu.pl (W.J.C.); aswlodarczyk@gumed.edu.pl (A.W.); mgaluszko@gumed.edu.pl (M.G.-W.)

**Keywords:** ketamine, treatment-resistant depression, cardiac safety, blood pressure, safety, tolerability

## Abstract

*Background and Objectives:* There is evidence for ketamine efficacy in treatment-resistant depression (TRD). Several safety and tolerability concerns arise that some psychotropic agents may provide blood pressure or/and heart rate alterations. The aim of this study is to review blood pressure measurements in course of the treatment with ketamine on treatment refractory inpatients with somatic comorbidities in the course of MDD and BP. *Materials and Methods:* The study population of 49 patients comprised MDD and BP subjects treated with ketamine registered in the naturalistic observational protocol of treatment-resistant mood disorders (NCT04226963). *Results:* The conducted analysis showed that among people suffering from hypertension there is a higher increase in systolic blood pressure (RR) after infusion 2 (*p* = 0.004) than among people who do not suffer from hypertension. Patients with hypertension have a higher increase in diastolic RR compared to those not suffering from hypertension (*p* = 0,038). Among the subjects with diabetes mellitus, significant differences occurred for infusions 2 (*p* = 0.020), 7 (*p* = 0.020), and 8 (*p* = 0.035) for heart rate (HR), compared to subjects without diabetes mellitus. A higher increase in diastolic RR was noted in the group of subjects suffering from diabetes mellitus (*p* = 0.010) compared to those who did not. In the hyperlipidemic patients studied, a significantly greater decrease in HR after infusion 5 (*p* = 0.031) and systolic RR after infusion 4 (*p* = 0.036) was noted compared to nonpatients. People after a stroke had significantly higher increases in diastolic RR after infusions 4 (*p* = 0.021) and 6 (*p* = 0.001) than those who did not have a stroke. Patients suffering from epilepsy had a significantly greater decrease in systolic RR after the 8th infusion (*p* = 0.017) compared to those without epilepsy. *Limitations:* The study may be underpowered due to the small sample size. The observations apply to inhomogeneous TRD population in a single-site with no blinding and are limited to the acute administration. *Conclusions:* This study supports evidence for good safety and tolerability profile for short-term IV ketamine use in TRD treatment. However, risk mitigation measures are to be considered in patients with metabolic and cardiovascular comorbidities.

## 1. Introduction

Several safety concerns arise with ketamine infusions in treatment-resistant depression (TRD). Sequalae observed are cardiovascular factors with a prominent focus on arterial hypertension [1,2,3,4]. As limited data are available from clinical trials and studies it appears that the treatment is overall safe [1,2,3]. Still, concerns arise for elderly patients and patients showing metabolic comorbidities [5]. In particular, excluded subjects with a history of stroke and epilepsy. Observational registries and protocols exist. Our study represents data for an ecological population with treatment-refractory mood disorders in an inhomogeneous group of patients. It may be hypothesized that somatic comorbidities are known to impact blood pressure (RR) and heart rate (HR), whether they affect RR in short-term inpatient treatment intravenous ketamine [6].

The literature data represents the correlation between oral, intranasal esketamine and intravenous ketamine [1,2,3,7], although safety and tolerability data contribute to safety knowledge as they correlate to up-to-date studies. It was hypothesized that patients with comorbidities may present higher RR, HR values as related to other patients. It was also hypothesized that some comorbidities may provide RR/HR alterations.

The aim of this study is to review blood pressure measurements in course of the treatment of resistant depression in patients with somatic comorbidities.

## 2. Methods and Population

The study population selection has been described in detail elsewhere [8,9]. Briefly, the study population comprises subjects enrolled in a naturalistic observational safety and efficacy registry protocol for ketamine infusions in TRD. Inpatients diagnosed with TRD-MDD and TRD-BP were included. Patients were interviewed by a clinician psychiatrist to establish the diagnosis according to the Diagnostic and Statistical Manual of Mental Disorders (DSM-5) criteria with Mini International Neuropsychiatric Interview (MINI). All participants exhibited treatment resistance for the current episode, defined as an inadequate response to two or more antidepressants (assessed by Massachusetts General Hospital Antidepressant Treatment Response Questionnaire—ATRQ) in the current MDD episode. Bipolar TRD was defined as a clinically unsatisfactory response to two approved and adequate interventions for bipolar depression [10]. Changes in concurrent psychotropics were allowed only after the follow-up period if the patients were inadequate responders to ketamine treatment. Psychometric assessments were scheduled per the study visit agenda. Subjects were defined as responders at a given time point if the percent improvement from baseline in MADRS total score was at least 50%, on the other hand, subjects were defined as remitters at a given time point if the MADRS total score was ≤10 points.

Only medically stable, adult inpatients aged 18–90 were enrolled to study. None of the patients suffered from psychotic symptoms in course of the disease. Some patients significantly affected by somatic illness continued current medication during ketamine treatment. The exclusion criteria included a history of uncontrolled medical conditions, a previous adverse reaction to ketamine, active substance use (verified by MINI, and also urine toxicology on screening and follow-up) including alcohol and cannabis, pregnancy, or breastfeeding.

All subjects gave written informed consent to participate in the study. The study was carried out in accordance with the latest version of the Declaration of Helsinki (NKBBN/172-674/2019) and Registered as NCT04226963.

## 3. Study Design: Ketamine Infusions

The study design was discussed in detail elsewhere [8,9]. Briefly, the study followed an observational design with patients continuing baseline standard-of-care medication receiving eight ketamine infusions over 4 weeks. Ketamine was dosed at 0.5 mg/kg and diluted into 0.9% saline solution administered over 40 min. Safety monitoring was assessed by the study clinician before, during, and post-infusion every 15 min up to 90 min post-infusion. Safety, including vital signs, mental status, and adverse incidents, were monitored and recorded at 15-min intervals. The ECG was carried out before every second infusion and 1 week after the last ketamine infusion. As a risk mitigation measure, guidance on RR monitoring on intranasal treatment days was provided to investigators (i.e., no dosing if predose systolic RR > 140 mmHg (>150 for age > 65 years) or diastolic RR > 90 mmHg; dose interruption if postdose systolic RR ≥ 200 mmHg (≥190 for age > 65 years) or diastolic RR ≥ 110 mmHg (≥100 for age > 65 years)); subjects with elevated RRs were referred to a specialist for evaluation and hypothetical treatment. Blood pressure and heart rate measurements were assessed after the patient had rested for at least 5 min and were measured supine, either with a certified, completely automated device at predose (0 min), and at 15, 40, 45, 60, 90 min.

## 4. Statistical Analysis

The primary outcome of interest was change in Systolic RR and Diastolic RR from preinfusion to highest postinfusion measurement explored post hoc in database registry. The analyses were conducted using statistical software the IBM SPSS Statistics 25.0. For sociodemographic variables and the occurrence of diseases and treatment, frequency analyses were carried out with Fisher’s exact test. When assessing the statistical significance between dichotomous groups, the Mann–Whitney *U* test was utilized. For within-subject and groups analyses the Kruskal–Wallis tests were conducted. α = 0.05 was adopted as the level of significance for the purposes of this analysis.

Due to the small sample size nonparametric tests were used and inequities among groups analyzed were used for the analysis for discrete and continuous variables.

## 5. Results

Demographic and clinical characteristics of the study population is presented in Table 1.

Using the Mann–Whitney U test, the mean-term blunt changes in HR and RR were compared with each other due to the use of a given type of medication. The results are presented in Table 2.

Overall, blood pressure elevations were observed in patients with comorbid: arterial hypertension, diabetes mellitus (DM), hyperlipidemia, and stroke history. Significant RR elevations were transient and observed at various points of time with no stable pattern, attributable to specific comorbidity or timepoint.

The conducted analysis showed that among people suffering from hypertension there is a higher increase in systolic RR after infusion 2 (*p* = 0.004) than among people who do not suffer from hypertension. A similar situation occurred for diastolic RR after one infusion (*p* = 0.038)—patients with hypertension have a higher increase in diastolic RR compared to those not suffering from hypertension.

Among the subjects with DM, significant differences occurred for infusions 2 (*p* = 0.020), 7 (*p* = 0.020) and 8 (*p* = 0.035) for HR. These subjects obtained a significantly higher increase in HR compared to subjects without DM. A higher increase in diastolic RR was noted in the group of subjects suffering from DM (*p* = 0.010) compared to those who did not.

In the hyperlipidemic patients studied, a significantly greater decrease in HR after infusion 5 (*p* = 0.031) and systolic RR after infusion 4 (*p* = 0.036) was noted compared to nonpatients.

People after stroke had significantly higher increases in diastolic RR after 4th infusion (*p* = 0.021) and 6th infusion (*p* = 0.001) than those who did not have a stroke.

Patients suffering from epilepsy had a significantly greater decrease in systolic RR after the 8th infusion (*p* = 0.017) compared to those without epilepsy.

## 6. Discussion

This study demonstrated good safety and tolerability profile of short-term ketamine infusions in TRD patients. The results point out to the risk of clinically significant RR elevation associated with cardiovascular and metabolic comorbidities. In TRD subjects with MDD and BP type I receiving IV ketamine across eight administrations, no clinically significant findings in RR and HR were observed.

The study replicates the observations in esketamine nasal spray [1,2,3], as well as a ketamine intravenous study conducted by McIntyre et al. [7], with the cardiac safety of ketamine in the depression treatment. When analyzing the different subgroups, it is apparent that hypertensive patients would have statistically significant increases in systolic and diastolic blood pressure compared to people without HA. However, these differences resolve at the end of monitoring with no sequelae.

There was evidence concluding that ketamine has a potential role in the treatment on somatic conditions such as acute pain, chronic pain, and headache, neurologic applications including neuroprotection and seizures, and alcohol and substance use disorders [6]. Moreover, ketamine has general impact on people with epilepsy, showing that ketamine appears to be effective and relatively safe for the control of multidrug-resistant, refractory status epilepticus [11], where adverse effects regarding increase of intraocular pressure, intracranial pressure, arrhythmias were rare. Ketamine was found to be beneficial in decreasing allodynia and hyperalgesia, as well as improving functional capabilities in post-stroke subjects [12]. There are also findings that ketamine has a favorable safety profile in obese patients, as these subjects often develop postoperative respiratory obstruction, hypoxia, and deep sedation, which are ketamine-preventable [13,14]. Laryngospasm was noted in <0.4% of adolescent-obese patients, as well as high IV ketamine doses (>2.5 mg/kg or >5.0 mg/kg total dose) led to amplified incidence of respiratory AEs, including brief (but harmless) episodes of apnea in those subjects. Lower doses (~1.5 mg/kg) did not induce these AEs, and were as effective as the larger doses when evaluating intraprocedural satisfaction in obese children [13]. In the above-mentioned somatic indications, no psychiatric comorbidities were noted, however major somatic ones were treated with the help of ketamine which appeared to be safe and an efficacious agent for treatment of somatic diseases. Though ketamine appears to be safe and effective, the reduction of elevated HR and RR was observed in time of the treatment with no sequelae nor harm. As transient increases in RR are a well-known feature of IV ketamine treatment [15], uncontrolled arterial hypertension is usually considered as a contraindication for ketamine application [16]. A multitude of clinical trials demonstrated the safety of ketamine administration in patients with concurrent conventional antidepressant medication [17]. Our findings report on ketamine safety and tolerability, supporting evidence for its safe use in populations with somatic comorbidities with particular caution on safety monitoring. A fear/anxiety is known to be associated with the elevated RR in ketamine administration. Recent evidence from esketamine nasal spray studies point to the increase RR incidence post esketamine nasal spray administration. That does not follow an anxiety habituation process contrasting to dissociation and sedation, both phenomena abating rapidly post initial drug administration [1].

Our study contributes to current literature, in particular bipolar TRD patients, as overall few studies focus on bipolar depression (0.7% of the studies addressed to BP population) [18]. It is of particular interest as these subjects receive mood stabilizers. To our best knowledge, this is the first study to report on the subjects with a comorbid recent history of stroke, hypercholesterolemia, DM, and epilepsy.

## 7. Limitation

The methodological strength of our study was to strengthen the point regarding the tolerability and general safety of the administration of the drug and that result being in support with some previous ketamine studies mentioned above.

The key study limitations include small sample size and lack of randomization with the intervention blinding. Thus, it may be underpowered, and its results shall be treated with caution as the observational design does not warrant comparative or causative conclusions. The observations apply to treatment-resistant depression and include both, MDD and bipolar depressed patients with a proportion of subjects diagnosed with somatic comorbidities and concomitant medication. Thus, the study population is inhomogeneous and represents subjects attending the tertiary-reference center. All safety and tolerability findings shall be replicated in a larger sample exposed to the intervention across the long-term study design. Although specific safety observations may apply to TRD patients presenting clinical response pattern, this study sample did not warrant such analysis that may be particularly worth consideration in future safety studies in larger samples.

## 8. Conclusions

This study supports evidence for good safety and tolerability profile for short-term IV ketamine use in TRD treatment in subjects with stable somatic illness. However, risk mitigation measures are to be considered in patients with metabolic and cardiovascular comorbidities. The abatement of elevated HR and RR was observed in time of the treatment with no sequelae nor harm. The side effects are mild or moderate, well-tolerated and transient as all of them cease within 4 h post administration. No exacerbations of cardiovascular, metabolic or seizure related conditions were observed.

## Figures and Tables

**Table 1:** Demographic and clinical characteristics of the study population is presented.

			*N*	Responder	Remitter	Nonresponder	*p*	V
Male, sex	(%)		21 (42.9)	6 (66.7)	2 (25.0)	13 (40.6)	0.229	0.26
age, years			50.02 (13.83)	53.11 (7.06)	42.88 (15.78)	50.94 (14.51)	0.336	0.00
BMI			27.92 (5.67)	28.00 (4.64)	26.50 (4.72)	28.25 (6.21)	0.613	0.02
ketamine treatment for:								
	MDD ^a^		35 (71.4)	8 (88.9)	5 (62.5)	22 (68.8)	0.475	0.19
	BP ^b^		14 (28.6)	2 (11.1)	5 (37.5)	7 (31.2)	0.485	0.18
comorbidities								
	arterial hypertension		16 (32.7)	6 (66.7)	3 (37.5)	7 (21.9)	0.037	0.37
		BP	4 (8.2)	1 (11.1)	2 (25.0)	1 (3.1)	0.052	0.66
		MDD	12 (24.5)	5 (55.6)	1 (12.5)	6 (18.8)	0.177	0.33
	diabetes mellitus		3 (6.1)	1 (11.1)	2 (25.0)	0 (0)	0.021	0.39
	hyperlipidemia		9 (18.4)	3 (33.3)	1 (12.5)	5 (15.6)	0.545	0.19
	post-stroke		3 (6.1)	1 (11.1)	0 (0)	2 (6.3)	0.731	0.14
	post-myocardial infarction		0 (0)	0 (0)	0 (0)	0 (0)	-	-
	epilepsy		6 (12.2)	0 (0)	3 (37.5)	3 (9.4)	0.060	0.36
	other		16 (32.7)	2 (22.2)	1 (12.5)	13 (40.6)	0.330	0.24

^a^ MDD—major depressive disorder, ^b^ BP—bipolar disorder.

**Table 2:** Using the Mann–Whitney U test, the mean-term blunt changes in HR and RR were compared with each other due to the use of a given type of medication.

		Non-HA (*n* = 33)				HA (*n* = 16)						
		M	SD	Me	IQR	M	SD	Me	IQR	Z	*p*	r
HR	infusion 1	−1.24	9.30	−1.69	9.92	−5.60	14.88	−0.72	13.64	−0.17	0.865	−0.02
	infusion 2	−1.65	8.27	−1.39	6.64	−0.67	7.15	−2.15	9.66	−0.31	0.759	−0.04
	infusion 3	−1.75	8.91	−1.00	11.81	−1.44	9.37	0.02	11.20	−0.09	0.932	−0.01
	infusion 4	−1.95	11.07	0.00	6.73	−4.75	10.27	−1.29	14.54	−0.80	0.424	−0.11
	infusion 5	−4.08	8.17	−2.15	11.47	1.95	10.54	0.00	11.62	−1.65	0.099	−0.24
	infusion 6	−2.08	9.42	−1.40	9.77	−1.89	8.17	−2.30	11.04	−0.14	0.887	−0.02
	infusion 7	−1.94	10.57	−2.74	13.34	0.72	7.83	0.00	12.97	−1.12	0.262	−0.16
	infusion 8	−3.57	13.02	−2.74	9.42	−0.85	10.46	−1.67	10.02	−0.57	0.566	−0.08
RR systolic	infusion 1	−0.84	6.78	0.00	7.45	0.77	14.32	0.00	11.69	−0.32	0.749	−0.05
	infusion 2	−2.21	6.68	−0.83	7.79	4.96	9.97	7.14	10.17	−2.88	0.004	−0.41
	infusion 3	−0.37	8.53	0.00	11.78	1.75	10.20	0.37	4.56	−0.70	0.481	−0.10
	infusion 4	−3.88	7.53	−1.64	8.19	−2.28	7.31	−3.32	12.39	−0.26	0.798	−0.04
	infusion 5	−0.27	9.83	−1.61	6.60	−0.10	8.41	0.00	10.26	−0.81	0.418	−0.12
	infusion 6	−0.37	6.54	0.00	8.69	57.51	227.25	0.00	6.97	−0.31	0.759	−0.04
	infusion 7	−1.15	7.94	0.00	9.77	−0.81	12.04	0.00	12.50	−0.12	0.904	−0.02
	infusion 8	−2.78	11.79	−3.82	12.85	−1.68	6.50	0.00	7.12	−0.75	0.454	−0.11
RR diastolic	infusion 1	−2.90	10.01	−2.78	10.04	6.12	14.69	2.63	16.69	−2.07	0.038	−0.30
	infusion 2	−1.89	7.30	0.00	6.05	4.42	12.00	4.22	17.34	−1.70	0.089	−0.24
	infusion 3	−2.15	13.75	0.00	15.69	−5.08	18.34	−0.63	20.99	−0.08	0.940	−0.01
	infusion 4	−0.53	10.43	0.00	14.47	−4.56	10.34	−0.63	15.61	−0.90	0.368	−0.13
	infusion 5	−1.00	9.96	−2.55	10.63	1.32	12.53	0.00	14.12	−0.75	0.456	−0.11
	infusion 6	−0.82	10.16	−0.63	8.38	2.23	7.22	0.00	8.34	−1.27	0.204	−0.18
	infusion 7	−1.67	11.48	−2.44	8.83	−2.13	6.10	0.00	4.15	−0.68	0.494	−0.10
	infusion 8	−1.51	9.78	0.00	13.13	−2.61	8.75	−1.25	10.77	−0.38	0.703	−0.05
		**Non-DM (*n* = 46)**				**DM (*n* = 3)**						
		**M**	**SD**	**Me**	**IQR**	**M**	**SD**	**Me**	**IQR**	**Z**	***p***	**r**
HR	infusion 1	−2.83	11.78	−1.65	12.63	−0.04	3.84	0.00	3.90	−0.50	0.617	−0.07
	infusion 2	−1.89	7.75	−2.02	6.74	7.08	3.55	8.33	−3.08	−2.32	0.020	−0.33
	infusion 3	−1.72	9.23	−0.50	12.00	−0.52	3.21	−1.37	3.23	−0.08	0.934	−0.01
	infusion 4	−2.90	11.06	0.00	8.38	−2.30	6.43	−1.19	9.21	0.00	1.000	0.00
	infusion 5	−2.13	9.60	−1.39	10.36	−1.13	5.96	1.16	7.89	−0.49	0.624	−0.07
	infusion 6	−2.10	9.06	−1.41	9.76	−0.88	8.27	−4.76	6.49	−0.09	0.932	−0.01
	infusion 7	−1.91	9.56	−1.67	10.92	10.10	1.75	9.09	−9.09	−2.32	0.020	−0.33
	infusion 8	−3.68	11.95	−2.78	8.96	9.63	9.33	5.00	−3.51	−2.10	0.035	−0.30
RR systolic	infusion 1	0.04	9.63	0.00	9.22	−5.78	12.38	0.00	20.00	−0.46	0.646	−0.07
	infusion 2	−0.24	8.62	0.00	11.69	6.62	3.05	7.14	−3.33	−1.70	0.088	−0.24
	infusion 3	0.11	9.27	0.00	10.58	3.55	4.30	2.33	0.00	−0.88	0.381	−0.13
	infusion 4	−3.16	7.37	−2.73	7.69	−6.32	9.25	−9.79	13.33	−0.88	0.381	−0.13
	infusion 5	−0.16	9.58	0.00	7.63	−1.04	2.45	0.00	3.85	−0.15	0.881	−0.02
	infusion 6	19.75	135.73	0.00	8.07	6.53	18.37	0.00	7.69	−0.04	0.966	−0.01
	infusion 7	−2.05	8.02	0.00	10.96	12.14	17.07	4.76	0.00	−1.69	0.092	−0.24
	infusion 8	−2.58	10.75	−3.36	9.90	−0.61	1.05	0.00	1.82	−0.56	0.574	−0.08
RR diastolic	infusion 1	0.00	12.46	0.00	9.62	0.64	12.80	−1.27	11.11	−0.10	0.917	−0.01
	infusion 2	−0.94	8.55	0.00	7.79	17.50	5.88	14.29	−13.92	−2.57	0.010	−0.37
	infusion 3	−3.56	15.36	−0.63	16.58	3.93	14.39	10.00	12.50	−0.94	0.348	−0.13
	infusion 4	−1.88	10.58	0.00	15.53	−1.44	10.56	0.00	12.64	−0.23	0.818	−0.03
	infusion 5	−0.51	11.01	−1.25	10.81	4.00	6.93	0.00	0.00	−0.92	0.358	−0.13
	infusion 6	−0.04	9.48	0.00	8.37	3.75	6.50	0.00	0.00	−1.05	0.292	−0.15
	infusion 7	−2.10	10.24	−2.38	6.74	1.78	3.08	0.00	0.00	−1.69	0.090	−0.24
	infusion 8	−1.55	9.04	0.00	11.64	−5.71	15.05	−7.14	20.00	−0.54	0.591	−0.08
		**Non-hCh. (*n* = 40)**				**hCh (*n* = 9)**						
		**M**	**SD**	**Me**	**IQR**	**M**	**SD**	**Me**	**IQR**	**Z**	***p***	**r**
HR	infusion 1	−2.00	12.13	0.00	11.38	−5.60	7.57	−1.69	8.32	−1.23	0.220	−0.18
	infusion 2	−1.21	8.26	−1.52	6.93	−1.84	6.14	0.00	9.51	−0.16	0.874	−0.02
	infusion 3	−1.91	8.88	−0.50	11.27	−0.49	9.78	−1.37	17.03	−0.32	0.747	−0.05
	infusion 4	−3.02	11.19	0.00	6.58	−2.14	9.34	−1.19	17.75	−0.12	0.907	−0.02
	infusion 5	−1.00	9.78	0.00	8.82	−6.70	5.62	−7.89	9.98	−2.15	0.031	−0.31
	infusion 6	−1.59	9.43	−1.39	9.83	−3.87	6.47	−4.76	9.37	−0.49	0.625	−0.07
	infusion 7	−0.27	9.35	−0.68	11.06	−4.39	11.26	−7.32	22.33	−0.98	0.328	−0.14
	infusion 8	−2.60	11.86	−2.70	9.28	−3.31	15.29	−5.36	22.41	−0.46	0.645	−0.07
RR systolic	infusion 1	0.81	9.79	0.00	7.67	−5.33	8.37	−1.34	14.03	−1.52	0.127	−0.22
	infusion 2	−0.03	8.43	0.00	11.05	1.13	9.41	3.33	9.47	−0.73	0.467	−0.10
	infusion 3	0.14	9.44	0.00	10.47	1.12	7.54	0.74	10.19	−0.56	0.579	−0.08
	infusion 4	−2.56	7.68	−1.16	8.15	−6.90	5.01	−7.69	5.65	−2.09	0.036	−0.30
	infusion 5	−0.65	8.73	0.00	8.89	1.69	11.82	0.00	5.39	−0.04	0.968	−0.01
	infusion 6	22.85	145.76	0.00	7.94	1.90	12.20	0.00	16.34	−0.16	0.874	−0.02
	infusion 7	−0.54	9.29	0.00	10.31	−3.13	9.95	1.57	16.77	−0.08	0.936	−0.01
	infusion 8	−2.57	11.16	−2.18	10.39	−1.68	3.03	−0.91	5.72	−0.23	0.815	−0.03
RR diastolic	infusion 1	0.31	12.97	0.00	10.43	−1.15	9.63	−4.55	15.64	−0.85	0.393	−0.12
	infusion 2	0.31	8.80	0.00	10.55	−0.20	12.73	−2.22	15.42	−0.49	0.624	−0.07
	infusion 3	−1.54	13.41	0.00	19.34	−10.05	21.34	−7.69	12.50	−1.20	0.230	−0.17
	infusion 4	−2.47	9.89	0.00	11.39	0.93	13.04	4.00	25.91	−1.01	0.311	−0.14
	infusion 5	0.25	11.33	0.00	12.20	−2.31	8.37	−2.25	7.14	−0.93	0.354	−0.13
	infusion 6	−0.17	9.82	0.00	8.45	1.78	6.96	0.00	10.59	−0.96	0.337	−0.14
	infusion 7	−1.01	10.45	−0.62	6.43	−5.28	6.84	−3.41	8.61	−0.85	0.393	−0.12
	infusion 8	−1.71	9.42	−0.75	11.68	−2.69	9.96	−0.63	12.89	−0.09	0.928	−0.01
		**Non-Stroke (*n* = 46)**				**Stroke (*n* = 3)**						
		**M**	**SD**	**Me**	**IQR**	**M**	**SD**	**Me**	**IQR**	**Z**	***p***	**r**
HR	infusion 1	−2.84	11.78	−1.53	12.54	0.17	3.23	−1.69	1.69	−0.42	0.677	−0.06
	infusion 2	−1.29	8.09	−1.52	7.19	−1.80	3.12	0.00	5.41	−0.28	0.782	−0.04
	infusion 3	−1.71	8.79	−1.15	10.93	−0.64	13.70	7.27	16.46	−0.71	0.478	−0.10
	infusion 4	−3.33	10.93	−0.60	10.19	4.29	4.94	7.14	1.41	−1.21	0.226	−0.17
	infusion 5	−1.85	9.47	−1.39	9.73	−5.47	8.35	−10.29	10.29	−0.66	0.509	−0.09
	infusion 6	−2.36	9.13	−2.94	9.71	3.04	0.32	3.23	−2.67	−1.68	0.092	−0.24
	infusion 7	−0.73	9.83	−0.68	12.82	−7.65	5.09	−7.65	11.25	−1.30	0.194	−0.19
	infusion 8	−2.65	12.35	−2.18	11.24	−5.06	-	−5.06	0.00	−0.59	0.554	−0.08
RR systolic	infusion 1	−0.53	10.05	−0.39	10.01	2.86	1.23	3.57	−1.45	−1.50	0.133	−0.21
	infusion 2	−0.14	8.67	0.00	11.69	5.13	4.44	7.69	0.00	−1.24	0.217	−0.18
	infusion 3	0.33	8.87	0.00	9.40	0.22	14.05	8.33	16.00	−0.42	0.676	−0.06
	infusion 4	−3.09	7.58	−1.95	8.99	−7.51	0.32	−7.69	7.69	−1.21	0.226	−0.17
	infusion 5	0.17	9.45	0.00	7.29	−5.90	3.55	−3.85	10.00	−1.64	0.101	−0.23
	infusion 6	20.05	135.73	0.00	7.91	1.99	11.62	8.70	11.43	−0.70	0.482	−0.10
	infusion 7	−0.81	9.45	0.00	10.74	−5.56	7.86	−5.56	11.11	−0.89	0.375	−0.13
	infusion 8	−2.50	10.49	−1.82	9.90	0.00	-	0.00	0.00	−0.50	0.620	−0.07
RR diastolic	infusion 1	0.54	12.57	0.00	10.20	−7.63	3.02	−5.88	11.11	−1.69	0.091	−0.24
	infusion 2	0.23	9.81	0.00	13.18	0.00	0.00	0.00	0.00	−0.19	0.847	−0.03
	infusion 3	−3.47	15.67	−1.27	18.17	2.50	4.33	0.00	0.00	−0.58	0.559	−0.08
	infusion 4	−2.75	10.02	0.00	15.32	11.97	8.14	16.67	−2.56	−2.31	0.021	−0.33
	infusion 5	0.07	11.07	0.00	11.23	−4.76	4.12	−7.14	7.14	−1.00	0.315	−0.14
	infusion 6	−0.81	8.69	0.00	6.48	15.27	4.10	12.90	−12.90	−2.60	0.009	−0.37
	infusion 7	−1.76	10.16	−1.81	6.64	−2.94	4.16	−2.94	5.88	−0.06	0.953	−0.01
	infusion 8	−2.07	9.39	−1.25	11.41	7.14	-	7.14	0.00	−1.20	0.231	−0.17
		**Non-Epileptic (*n* = 43)**				**Epileptic (*n* = 6)**						
		**M**	**SD**	**Me**	**IQR**	**M**	**SD**	**Me**	**IQR**	**Z**	***p***	**r**
HR	infusion 1	−2.39	11.57	−1.45	10.65	−4.58	11.35	−4.01	18.91	−0.64	0.522	−0.09
	infusion 2	−2.04	7.84	−2.27	6.94	3.69	6.39	0.70	12.69	−1.89	0.059	−0.27
	infusion 3	−1.67	9.02	0.00	10.89	−1.46	9.40	−1.48	17.69	−0.15	0.879	−0.02
	infusion 4	−3.47	10.46	−1.19	6.43	1.51	13.15	7.44	23.59	−1.50	0.135	−0.21
	infusion 5	−2.46	9.52	−1.42	9.89	0.67	8.44	1.09	12.20	−0.92	0.358	−0.13
	infusion 6	−1.67	8.99	−1.44	9.60	−4.50	8.89	−3.25	15.05	−0.56	0.575	−0.08
	infusion 7	−0.49	10.08	−0.68	12.36	−4.42	7.02	−4.67	12.67	−0.99	0.323	−0.14
	infusion 8	−2.21	12.69	−1.67	12.32	−5.61	9.17	−3.11	10.18	−0.72	0.472	−0.10
RR systolic	infusion 1	−0.26	10.10	0.00	7.80	−0.72	7.65	0.76	13.43	−0.24	0.807	−0.03
	infusion 2	0.23	8.69	0.00	11.91	−0.15	8.07	2.27	12.84	−0.14	0.888	−0.02
	infusion 3	0.93	8.94	0.00	9.17	−4.05	9.47	−4.43	14.55	−1.44	0.151	−0.21
	infusion 4	−2.74	6.51	−1.64	8.70	−7.80	12.07	−4.92	12.84	−0.81	0.419	−0.12
	infusion 5	−0.73	9.20	−0.77	6.02	3.38	9.97	4.55	15.77	−1.14	0.255	−0.16
	infusion 6	21.89	140.43	0.00	8.56	−1.83	7.15	0.41	8.47	−0.14	0.888	−0.02
	infusion 7	−0.14	9.33	0.00	11.08	−6.38	8.22	−4.55	13.43	−1.64	0.102	−0.23
	infusion 8	−1.35	10.63	0.00	7.97	−8.97	5.63	−8.12	9.87	−2.38	0.017	−0.34
RR diastolic	infusion 1	−0.73	11.65	−1.20	8.85	5.56	16.76	5.83	22.59	−1.65	0.099	−0.24
	infusion 2	−0.05	9.54	0.00	9.28	2.06	9.87	0.00	15.48	−0.58	0.562	−0.08
	infusion 3	−1.73	15.42	0.00	17.79	−12.94	10.33	−12.14	19.47	−2.08	0.038	−0.30
	infusion 4	−1.29	9.75	0.00	16.07	−5.86	15.25	−6.53	19.52	−0.86	0.390	−0.12
	infusion 5	−1.19	10.02	−0.63	10.34	6.50	14.55	2.00	21.38	−1.22	0.223	−0.17
	infusion 6	0.72	8.96	0.00	8.07	−3.50	11.80	−1.88	19.67	−0.61	0.539	−0.09
	infusion 7	−2.53	7.82	0.00	6.64	2.45	18.87	−3.28	15.68	−0.49	0.626	−0.07
	infusion 8	−0.63	9.10	0.00	12.29	−9.17	8.22	−9.35	14.77	−1.91	0.057	−0.27

HA—arterial hypertension; DM—diabetes mellitus; hCh—hypercholersterolemia.

## Data Availability

The data presented in this study are available on request from the corresponding author.
